# Moderate Continuous Aerobic Exercise Training Improves Cardiomyocyte Contractility in Β_1_ Adrenergic Receptor Knockout Mice

**DOI:** 10.5935/abc.20180025

**Published:** 2018-03

**Authors:** Aurora Corrêa Rodrigues, Antônio José Natali, Daise Nunes Queiroz da Cunha, Alexandre Jayme Lopes Dantas Costa, Anselmo Gomes de Moura, Miguel Araújo Carneiro-Júnior, Leonardo Bonato Félix, Patrícia Chakur Brum, Thales Nicolau Prímola-Gomes

**Affiliations:** 1 Universidade Federal de Viçosa (UFV), Viçosa, MG - Brazil; 2 Universidade de São Paulo (USP), São Paulo, SP - Brazil

**Keywords:** Heart Failure, Exercise, Myocardial Contraction, Myocytes, Cardiac, Adrenergic beta 1 Receptor Antagonists, Mice

## Abstract

**Background:**

The lack of cardiac β_1_-adrenergic receptors (β_1_-AR) negatively affects the regulation of both cardiac inotropy and lusitropy, leading, in the long term, to heart failure (HF). Moderate-intensity aerobic exercise (MCAE) is recommended as an adjunctive therapy for patients with HF.

**Objective:**

We tested the effects of MCAE on the contractile properties of left ventricular (LV) myocytes from β_1_ adrenergic receptor knockout (β_1_ARKO) mice.

**Methods:**

Four- to five-month-old male wild type (WT) and β_1_ARKO mice were divided into groups: WT control (WTc) and trained (WTt); and β_1_ARKO control (β_1_ARKOc) and trained (β_1_ARKOt). Animals from trained groups were submitted to a MCAE regimen (60 min/day; 60% of maximal speed, 5 days/week) on a treadmill, for 8 weeks. P ≤ 0.05 was considered significant in all comparisons.

**Results:**

The β_1_ARKO and exercised mice exhibited a higher (p < 0.05) running capacity than WT and sedentary ones, respectively. The β_1_ARKO mice showed higher body (BW), heart (HW) and left ventricle (LVW) weights, as well as the HW/BW and LVW/BW than WT mice. However, the MCAE did not affect these parameters. Left ventricular myocytes from β_1_ARKO mice showed increased (p < 0.05) amplitude and velocities of contraction and relaxation than those from WT. In addition, MCAE increased (p < 0.05) amplitude and velocities of contraction and relaxation in β_1_ARKO mice.

**Conclusion:**

MCAE improves myocyte contractility in the left ventricle of β_1_ARKO mice. This is evidence to support the therapeutic value of this type of exercise training in the treatment of heart diseases involving β_1_-AR desensitization or reduction.

## Introduction

Chronic sympathetic hyperactivity resulting from altered autonomic nervous system balance is common in many cardiovascular disease states, ending up in heart failure (HF), and is related to a higher incidence of morbidity and mortality.^1,2^ Such hyperactivity is paralleled by a decrease in b-adrenergic receptors (b-AR) density and desensitization of the remaining b-AR, thus leading to a reduced cardiac contractile response to b-AR activation.^3^ In this framework, β_1_-AR, predominant in the heart, is selectively reduced, resulting in a modified ratio of β_1_ to β_2_ subtypes,^4^ and β_2_-AR are markedly coupled to inhibitory G protein. Consequently, inasmuch as the β_1_-AR phosphorylates several Ca^2+^ regulatory proteins involved in cardiomyocyte excitation-contraction coupling,^5-7^ cardiac chronotropism, inotropism and lusitropism are impaired under adrenergic stimulation.^8^

Exercise training in cardiac rehabilitation is very important in several cardiovascular diseases, including chronic HF.^9^ Continuous moderate-intensity aerobic exercise (MCAE) is, at present, the best-established form of exercise for this population because of its efficacy and safety.^10^ For example, aerobic exercise training recovers the resting autonomic balance in HF patients by reducing the resting sympathetic nerve activity,^11^ and restoring the parasympathetic tone to the heart.^12,13^ In the myocardium, aerobic exercise training increases stroke volume and, hence, cardiac output in patients^14,15^ and in animal models of HF,^8^ although some studies failed to confirm such benefits.^11,12^ At the cellular level, studies on animal models for sympathetic hyperactivity have demonstrated aerobic exercise training improves the net balance of cardiac Ca^2+^ handling proteins either alone^8,16^ or in combination with b-blockers.^17^ Nevertheless, whether MCAE training affects mechanical properties of single myocytes in a heart lacking β_1_-AR remains to be elucidated.

Therefore, the aim of this study was to test the effects of an MCAE program on mechanical properties of single left ventricular (LV) myocytes in β_1_AR knockout (β_1_ARKO) mice. We hypothesized that MCAE training positively affects mechanical properties of LV myocytes from β_1_ARKO mice.

## Methods

### Experimental animals

A cohort of 4- to 5-month-old male wild type (WT) and congenic β_1_ARKO mice in the C57Bl6/J genetic background were studied. Mice were maintained in cages under a 12:12-h light-dark cycle in a temperature-controlled room (22ºC), with free access to water and standard rodent diet. WT and β_1_ARKO mice were randomly assigned into one of the following groups by using the simple random sampling: WT control (WTc), WT trained (WTt), β_1_ARKO control (β_1_ARKOc) and β_1_ARKO trained (β_1_ARKOt). The sample size was defined by convenience. All groups initiated the experimental period with eight animals, however, during the cardiomyocyte isolation procedure, some animals/hearts were lost. Thus, the final number of animals in each group is specified in figures and table. Body weight (BW) was measured every week. The experimental protocols were approved by the Ethics Committee for Animal Use at the Viçosa Federal University (protocol #59/2012) in accordance with the Guide for the Care and Use of Laboratory Animals/2011.

### Exercise training protocol and graded treadmill exercise test

MCAE was performed on a motor treadmill (Insight Equipamentos Científicos, Brazil) 5 days/week (Monday to Friday), 60 min/day, for 8 weeks. Over the first week, the duration and running speed of exercise were progressively increased from 10 minutes and 10% of the maximal speed until 60 minutes and 60% of the maximal speed, achieved during a graded treadmill exercise test. At the end of the fourth week of aerobic exercise training, graded treadmill exercise tests were repeated to readjust the running speed. This intensity was maintained during the rest of the training period. During the training period, animals from the untrained groups were handled every day and subjected to a short period of mild exercise (5 min, 0% grade, 5 m/min, 3 days/week). The exercise capacity estimated by total distance run was evaluated using a graded treadmill exercise protocol for mice (Panlab/Harvard Apparatus, Spain), as described previously.^18^ Briefly, after being adapted to the treadmill for 1 week (10 min/day, 0% grade, 0.3 km/h), mice were placed in the exercise streak and allowed to acclimatize for at least 30 minutes. The graded treadmill exercise test began at 6 m/min with no grade and increased by 3 m/min every 3 minutes until fatigue, which was defined as when the test was interrupted because the animals could no longer keep pace with the treadmill speed. The graded treadmill exercise test was performed in WT and b1ARKO untrained and exercise-trained groups before and after the exercise training period.

### Cardiomyocyte isolation

Forty-eight hours after the last exercise training session, mice were weighed and killed by decapitation, and their hearts were removed quickly. Left ventricular myocytes were enzymatically isolated as described previously.^19^ Briefly, hearts were mounted onto a Langendorff system and perfused with calcium-free HEPES-Tyrode solution for 6 minutes with the following composition (in mM): 130 NaCl, 1.43 MgCl_2_, 5.4 KCl, 0.4 NaH_2_PO_4_, 0.75 CaCl_2_, 25 HEPES, 22 glucose, 0.01 µg/ml insulin, 0.1 EGTA, pH 7.4, at 37ºC. Afterwards, the hearts were perfused for 7-10 minutes with a solution containing 1 mg/ml collagenase type II (Worthington, USA) and CaCl_2_ (0.8 µM). The digested heart was then removed from the perfusion apparatus and the heart and left ventricle were carefully weighed. Left ventricle was cut into small pieces and placed into conical flasks with collagenase-containing solution. The cells were dispersed by agitating the flasks for periods of 3 minutes at 37ºC. Single cells were separated from the non-dispersed tissue by filtration. The resulting cell suspension was centrifuged and resuspended in HEPES-Tyrode solution containing CaCl_2_ (2.5 and 5 µM, subsequently). The isolated cells were stored in HEPES-Tyrode solution containing 10 µM CaCl_2_ at room temperature until use. Only calcium-tolerant, quiescent, rod-shaped cardiomyocytes showing clear cross-striations were studied. The isolated cardiomyocytes were used within 2-3 hours of isolation.

### Cell contractility measurement

Cell contractility was evaluated as described previously.^20^ Briefly, the isolated cells were placed in a chamber with a glass coverslip base mounted onto the stage of an inverted microscope (Nikon Eclipse, TS100). The chamber was perfused with HEPES-Tyrode solution plus 10 *µ*M CaCl_2_ at 37ºC. Steady-state 1-Hz contractions were elicited via platinum bath electrodes (Myopacer, Field Stimulator, IonOptix) with 5-ms voltage pulses and an intensity of 40 V. The cells were visualized on a personal computer monitor with a NTSC camera (MyoCam, IonOptix) in partial scanning mode. The image was used to measure cell shortening (our index of contractility) in response to electrical stimulation using a video motion edge detector (IonWizard, IonOptix). The cell image was sampled at 240 Hz. Cell shortening was calculated from the output of the edge detector using an A/D converter (IonOptix, Milton, MA). Cell shortening (expressed as percentage of resting cell length) and the velocities of shortening and relaxation were calculated.

### Statistics

Data were subjected to Shapiro-Wilk or Kolmogorov-Smirnov normality tests as appropriate. Paired *t* test was used to compare initial and final BW in each group. The comparisons among groups of the values of BW, heart weight (HW), left ventricular weight (LVW) and ratios, as well as cell contraction were made using a two-way ANOVA followed by Tukey test using software SigmaPlot®, 12.5 version (Systat Software, San Jose, CA). Data are presented as means ± SD. A statistical significance level of 5% was adopted. Numbers of mice, hearts, and myocytes used are given in the relevant table and figure legends.

## Results

Table 1 shows BW and LVW. The initial BW of β_1_ARKO animals was higher as compared to their respective control WT animals. As expected, the final BW of each group was higher, compared to their respective initial BW. The final BW was higher (p < 0.05) in β_1_ARKO (β_1_ARKOc + β_1_ARKOt), compared to WT mice (WTc + WTt). However, the final BW was not affected (p > 0.05) by the MCAE. Likewise, HW was higher in β_1_ARKO than in WT mice, but no effect of MCAE was observed (p > 0.05). Regarding LVW, β_1_ARKO presented higher values than WT mice; nevertheless, no effect of MCAE was found (p > 0.05). As for the ratios, β_1_ARKO mice presented higher HW to BW ratio than WT mice. However, it was not affected by MCAE (p > 0.05). The LVW to BW ratio was higher in β_1_ARKO mice, compared to WT mice, but there was no effect of MCAE.

**Table 1:** Body and left ventricular weights

	WTc (n = 7)	WTt (n = 6)	β_1_ARKOc (n = 7)	β_1_ARKOt (n = 6)
Initial BW, g	27.43 ± 2.46	26.50 ± 2.45	33.86 ± 2.46	32.67 ± 2.23
Final BW, g	29.86 ± 2.64*	28.67 ± 2.64*	37.14 ± 2.64*	34.33 ± 2.55*
HW, mg	231.00 ± 37.57	226.00 ± 37.48	302.00 ± 37.57	317.00 ± 37.48
LVW, mg	146.00 ± 20.82	141.00 ± 20.82	184.00 ± 20.82	194.00 ± 20.82
HW/BW, mg/g	7.73 ± 0.85	7.86 ± 0.86	8.12 ± 0.85	9.22 ± 0.86
LVW/BW, mg/g	4.89 ± 0.48	4.94 ± 0.49	4.96 ± 0.48	5.66 ± 0.49

Values are means ± SD; WTc: wild-type control; WTt: wild-type trained; β_1_ARKOc: knockout β_1_-ARs control; β_1_ARKOt: knockout β_1_-ARs trained; BW: body weight; HW: heart weight; LVW: left ventricular weight; N: number of animals;

* p < 0.05 vs. initial BW within the same group. Statistical differences were determined by paired t test.

**Figure 1:**
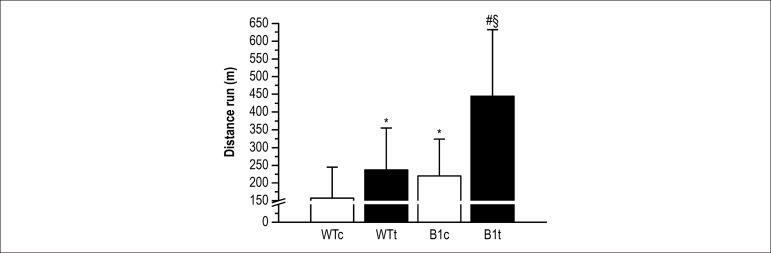
Total distance run. Values are means ± SD of 8 mice in each group. *p < 0.05 vs. WTc group; §p < 0.05 vs. WTt group; #p < 0.05 vs. β1ARKOc group.

Figure 1 shows the physical capacity. β_1_ARKO animals (β_1_ARKOc + β_1_ARKOt) exhibited a longer running distance, compared to WT animals (WTc + WTt). In addition, trained animals presented a longer running distance, compared to their respective controls.

**Figure 2:**
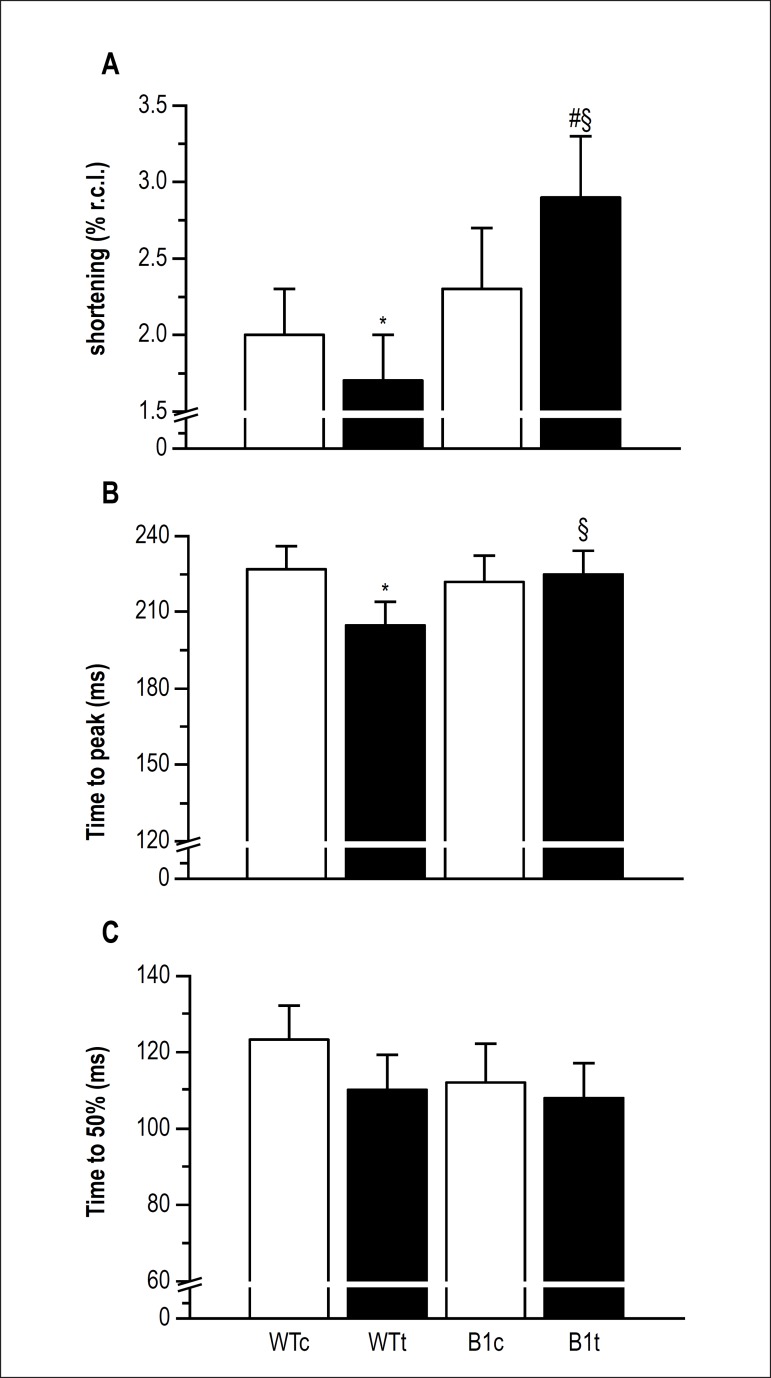
Cell contractility. A) Shortening. B) Velocity of shortening. C) Velocity of relaxation. WTc, wild-type control (n = 7; N = 14-39 cells from each mouse); WTt, wild-type trained (n = 6; N = 8-27 cells from each mouse); β1ARKOc, knockout β1-AR control (n = 7; N = 24-31 cells from each mouse); β1ARKOt, knockout β1-AR trained (n = 6; N = 17-29 cells from each mouse). Values are means ± SD.*p < 0.05 vs. WTc group; §p < 0.05 vs. WTt group; #p < 0.05 vs. β1ARKOc group.

The contractile properties of single LV myocytes are presented in Figure 2. β_1_ARKO myocytes (β_1_ARKOc + β_1_ARKOt) had higher shortening amplitude that WT cells (WTc + WTt). The amplitude of shortening in β_1_ARKOt myocytes was higher, compared to β_1_ARKOc and WTt cells; and in WTc cells, compared to WTt cells (Figure 2A). Regarding the contractile time course, β_1_ARKOc myocytes exhibited higher velocity of shortening than WTc cells. In addition, β_1_ARKOt myocytes exhibited higher velocity of shortening than β_1_ARKOc and WTt cells (Figure 2B). As for the velocity of relaxation, β_1_ARKOc myocytes exhibited higher values than WTc cells. Moreover, β_1_ARKOt myocytes exhibited higher velocity of relaxation than β_1_ARKOc and WTt cells (Figure 2C).

## Discussion

In this study, we tested the effects of MCAE on mechanical properties of LV myocytes from β_1_ARKO mice. The main finding was that MCAE increased the amplitude of shortening and velocities of shortening and relaxation in β_1_ARKO mice myocytes.

The initial and final BWs were higher in β_1_ARKO than in WT mice. Similar results have been observed elsewhere.^21^ b-AR activation in adipose tissue leads to cyclic adenosine monophosphate (cAMP) production, which activates protein kinase A (PKA) and stimulates lipolysis. Even though β_3_-AR is the predominant receptor in rodent adipose tissue, mice overexpressing β_1_-AR exhibit increased adipocyte lipolytic activity.^22^ Therefore, β_1_ARKO mice may have reduced lipolysis, which would influence the amount of body fat and, consequently, BW.^23^ Nevertheless, our MCAE did not affect the final BW. Regarding HW, β_1_ARKO mice exhibited heavier hearts and left ventricles than WT mice, as well as higher HW to BW and LVW to BW ratios. Our MCAE, nevertheless, did not modify these cardiac parameters. Exercise-induced cardiac hypertrophy in WT mice has been demonstrated elsewhere;^24-26^ nevertheless in β_1_ARKO mice, as far as we know, no data have been reported.

We observed that trained mice (WTt and β_1_ARKOt) showed longer total running distance than their respective controls (WTc and β_1_ARKOc). This MCAE-induced increase may be associated with cardiovascular adaptations, which are known features of aerobic exercise training.^27^ Previous studies using the same aerobic exercise training protocol also observed increased exercise capacity in trained animals.^8,17^ Specifically, the β_1_ARKO groups showed longer total running distance than the WT groups. It is known that sympathetic activation during aerobic exercise promotes glycogenolysis by b-AR pathway.^28,29^ Probably, the β_1_ARKO mice have compensatory mechanisms in the skeletal muscle, such as modified β_2_ and α_1_ adrenergic signaling pathways, which could improve glycogenolysis, gluconeogenesis, insulin-independent glucose uptake and lipolysis in the skeletal muscles.^30^ These compensatory mechanisms may have led to increased exercise performance in β_1_ARKO mice. However, inasmuch as this issue is not the focus of this study, further investigations are needed to test the hypothesis that β_1_ARKO mice increase exercise performance by altering β_2_ and α_1_ adrenergic signaling pathways.

Although myocytes from β_1_ARKO mice had a higher amplitude of shortening than cells from WT mice, an independent factor effect, LV myocytes from β_1_ARKOc and WTc groups had similar contractile properties. Although β_1_AR is the predominant adrenergic receptor subtype expressed in the heart in terms of density and modulation of cardiac contraction,^31,32^ its deletion had little impact on resting cardiac function, but had significant effects on cardiac function after b-agonist stimulation.^33^ Other studies did not observe changes in cardiomyocyte contractility upon loss of β_1_-AR^34^ or β_1/2_-AR under basal conditions.^35^ Therefore, the similarity between β_1_ARKOc and WTc groups suggests that β_1_-AR has little impact on the contractile properties of cardiomyocytes under basal conditions.

More important, the MCAE program increased the amplitude of shortening of LV myocytes from β_1_ARKO mice. The MCAE may have triggered two compensatory mechanisms in the heart of β_1_ARKO mice. First, an increase in α_1_-ARs signaling is common under situations of β_1_-ARs desensitization when the reduction of β_1_-adrenergic signaling is compensated by an increase in α_1_-adrenergic signaling pathway, which could help preserve cardiac function.^36^ Although not evaluated here, an increased inotropic responsiveness of rat cardiomyocytes via α_1_-AR stimulation was found as an adaptation to aerobic exercise training.^37,38^ Moreover, the potential therapeutic role of α_1_-ARs to maintain normal cardiac function, especially in terms of commitment of the β_1_-adrenergic signaling pathway, has been proposed in previous studies.^37-40^ Second, MCAE may have reduced the responsiveness of β_2_-AR in myocytes of β_1_ARKO mice. When β_2_-AR coupling to G_i_ protein is reduced, the inhibitory effect of the receptor to adenylate cyclase activation is also reduced,^5^ which causes an increased cAMP production and phosphorylation of proteins involved in cardiomyocyte excitation-contraction coupling.^6^

The time courses of β_1_ARKO LV myocyte contraction and relaxation were also improved by MCAE, indicating enhanced systolic and diastolic functions. The Ca^2+^ regulatory proteins modulate cardiomyocyte mechanical properties. While faster myocyte contraction is associated with increased density and or activity of L-type Ca^2+^ channels and RyR_2_, quicker relaxation is dependent on the increased activity and or density of SERCA2a, PLB and NCX.^6^ Although not measured in the present study, MCAE may have improved the net balance of cardiac Ca^2+^ handling proteins in β_1_ARKO mice. Such adaptations have been demonstrated previously in a different model for sympathetic hyperactivity.^8,16^ In addition, endurance-exercise training may have reduced the b/a-MHC ratio,^20^ which would also help explain the increased velocities of LV myocyte contraction and relaxation.

In recent years, high-intensity interval training (HIIT) has emerged as the method that leads to significant benefits to cardiac function. For instance, mice submitted to HIIT presented higher cardiomyocyte contractile function by increasing the expression and activity of calcium cycle regulatory proteins, as compared to those submitted to MCAE.^41-43^ Thus, it is possible that cardiomyocytes from β_1_ARKO mice might be more responsive to HIIT. However, in the present study, we chose the MCAE because the effects of such exercise protocol on the single cardiomyocyte contractility in β_1_ARKO mice are not known. We believe that future studies using HIIT would provide interesting findings in this animal model.

This study has limitations. First, we used global KO mice and systemic alterations confounding the exercise effects may have occurred, thus these results have to be interpreted with caution. Second, although WTt animals had improved their exercise capacity, unexpectedly their LV myocytes presented lower cell shortening than WTc mice. This finding really intrigued us, and, unfortunately, we cannot explain it.

## Conclusion

In conclusion, MCAE training improves myocyte contractility in the left ventricle of β_1_ARKO mice. This finding has potential clinical implications and supports the therapeutic value of this type of exercise training in the treatment of heart diseases involving β_1_-AR desensitization or reduction.
